# A Plasmacytoid Dendritic Cells-Type I Interferon Axis Is Critically Implicated in the Pathogenesis of Systemic Lupus Erythematosus

**DOI:** 10.3390/ijms160614158

**Published:** 2015-06-23

**Authors:** Ji-Min Kim, Sung-Hwan Park, Ho-Youn Kim, Seung-Ki Kwok

**Affiliations:** 1 Division of Rheumatology, Department of Internal Medicine, Dongsan Medical Center, Keimyung University School of Medicine, Daegu 700-712, Korea; E-Mail: okjimin@hanmail.net; 2 Division of Rheumatology, Department of Internal Medicine, Seoul St. Mary’s Hospital, College of Medicine, The Catholic University of Korea, Seoul 137-701, Korea; E-Mail: rapark@catholic.ac.kr; 3 Division of Rheumatology, Department of Internal Medicine, Konkuk University Medical Center, Seoul 143-729, Korea; E-Mail: ho0919@gmail.com

**Keywords:** systemic lupus erythematosus, dendritic cells, type I interferon

## Abstract

Systemic lupus erythematosus (SLE) is a prototypic autoimmune disease that is characterized by the generation of immune responses to various nuclear components. Impaired clearance of apoptotic cells and loss of tolerance to self-antigens are involved both in the initiation and in the propagation of the disease. Dendritic cells (DCs) are key factors in the balance between autoimmunity and tolerance and play a role linking innate and adaptive immunity. DCs, particularly plasmacytoid DCs (pDCs), are the main source of type I interferon (IFN) cytokines, which contribute to the immunopathogenesis of SLE. There is accumulating evidence that pDCs and type I IFN cytokines take the leading part in the development of SLE. In this review, we discuss recent data regarding the role of pDCs and type I IFN cytokines in the pathogenesis of SLE and the potential for employing therapies targeting against aberrant regulation of the pDC-type I IFN axis for treating SLE.

## 1. Introduction

Systemic lupus erythematosus (SLE) is an autoimmune disease that affects multiple organs and has significant mortality. The etiology of SLE includes both genetic and environmental factors, but remains poorly understood. Various immunologic abnormalities contribute to the pathogenesis of SLE. These include disturbed clearance of apoptotic cells, loss of tolerance to self-antigens, aberrant activation of T and B cells, altered cytokine profiles, and pathogenic autoantibody production. The autoantibodies, which are serologic hallmarks of SLE, are mostly directed against nuclear antigens including double-stranded DNA (dsDNA), small nuclear ribonucleoproteins (snRNPs), and nucleosomes although autoantibodies are also directed against antigens located in the cytoplasm, those on the cell surface, and those secreted by the cell [1,2]. Numerous factors including abnormal apoptosis of immune cells, undisposed apoptotic cell debris, and aberrant interactions between hyperactive B and T cells are associated with autoantibody production, which can lead to the formation of immune complexes (ICs) in patients with SLE [3]. During the process of unraveling this complicated immunologic problem, clues that dendritic cells (DCs) and type I interferon (IFN) play a critical role in the pathogenesis of SLE have emerged over the past few decades. This review will focus on both the normal physiologic roles of DCs in the human immune system and our current understanding of the role of DCs and type I IFN in SLE. In addition, we will discuss recent data concerning new therapeutic approaches focused on DCs and type I IFN in SLE.

## 2. DCs and Immune Tolerance

### 2.1. DC Subsets & Characteristics

DCs were first identified by Steinman and Cohn based on their characteristic stellate morphology. Myeloid DCs (mDCs) and plasmacytoid DCs (pDCs) are two major subtypes of DCs [4]. Although DCs share the capacity to present antigens and regulate T and B cells, there are several distinctions between mDCs and pDCs. mDCs, which have a classical finger-like projection morphology, are professional antigen presenting cells (APCs) bearing various Toll-like receptors (TLRs) including TLR 1, 6, 8 and 10 [5]. Human mDCs are characterized by high expression levels of CD11c, CD1a and HLA-DR [6]. Unlike mDCs, pDCs, which have an appearance similar to that of plasma cells, release copious amounts of type I IFNs following activation and induce differentiation of B cells into antibody-producing plasma cells [7]. Although the pDC population is less than 1% of human peripheral blood mononuclear cells, they produce the majority of type I IFNs in the human body upon exposure to viral particles [8]. Human pDCs, which are negative for CD11c and CD1a, express IL-3 receptor (CD123), blood dendritic cell antigen (BDCA)-2 (CD303), neuropilin-1 (CD304 or BDCA-4), and relatively low level of HLA-DR [9]. pDCs can recognize pathogens through TLRs, such as TLR7 and TLR9 [10].

### 2.2. DCs as Guards against Autoimmunity

DCs, which link innate and adaptive immunity, serve as regulators of the immune system. They potentially promote immune tolerance and, on the contrary, might also initiate autoimmune reactions, depending on their circumstances. Steady-state DCs with an immature phenotype are essential for the induction and maintenance of peripheral immune tolerance. Although there are conflicting studies about the influence of the status of DCs on the maintenance of immune tolerance, activated DCs are universally considered to lose their ability to stabilize autoreactive T cells and induce regulatory T (Treg) cells [11,12].

At steady state, DCs contribute to immune tolerance. In the thymus, thymic DCs, along with medullary thymic epithelial cells (mTECs), present many tissue-specific self-antigens to developing thymocytes. Peripheral DCs also migrate into the thymus and present peripheral self-antigens to developing thymocytes to induce negative selection of autoreactive T cells [13]. DCs also promote the generation of Treg cells in the thymus [14]. In addition, DCs are considered to play an essential role in peripheral tolerance. In the normal milieu, antigen presentation by DCs induces an anergic state in effector T cells by overexpressing inhibitory molecules such as programmed cell death protein 1 (PD1) and cytotoxic T lymphocyte antigen 4 (CTLA4) on T cells [15]. Ligation of PD1 and PD1 ligand 1 (PDL1) or CTLA4 and CD80/86 is important for the induction of T cell tolerance [15]. DCs also seem to contribute to the induction and the maintenance of Treg cells in the periphery [16,17]. In the presence of transforming growth factor-β (TGF-β) and retinoic acid, DCs can induce Treg cells from naïve CD4+ T cells [12,18]. Expression of costimulatory molecules, CD80 and/or CD86 on DCs is also necessary for the maintenance of the number of Treg cells [19].

## 3. The Role of DCs in SLE

It has been widely demonstrated that dysregulations of both the innate and adaptive immune systems contribute to the pathogenesis of SLE. DCs, which link innate and adaptive immunity, are implicated in the development of SLE. Removal of DCs in MRL-Fas*^lpr^* mice, a lupus-prone mouse model, ameliorated the disease and decreased T cell expansion as well as IgG/IgM autoantibody formation [20]. The specific ablation of CD95 (a death receptor also known as Fas) in DCs induces several disease manifestations such as production of antinuclear antibody and hyperimmunoglobulinemia in C57BL/6 mice [21]. DCs from SLE patients exhibit distinctive expression of co-stimulatory and inhibitory surface markers [22]. Among numerous previous studies that demonstrate the role of DCs in the development of SLE, pDCs and their product, type I IFN, are especially highlighted in the immunopathogenesis of SLE.

### 3.1. pDCs in SLE

Recent studies have provided circumstantial evidence that pDCs play an important role in the pathogenesis of SLE. Several reports discuss the altered population of pDCs in patients with SLE. Migita *et al.* demonstrated that circulating BDCA-2-positive pDCs are reduced in patients with SLE [23]. Fiore *et al.* and Tucci *et al.* reported that the number of pDCs in the blood of SLE patients is decreased, while pDC infiltrations are increased in the kidney tissues of patients with lupus nephritis, suggesting that activated pDCs might have migrated to the target organ [24,25]. Accumulation of pDCs in skin lesions has also been confirmed in SLE patients [26,27]. Phenotypic and functional changes of pDCs were investigated in SLE. Kwok *et al.* demonstrated that pDCs in SLE patients are exhausted and show a diminished response upon TLR9 stimulation [28]. Nie *et al.* revealed that bone marrow-derived pDCs of SLE patients have high expression levels of CD40 and CD86 and have a strong capacity to induce T cell proliferation [29]. Low expression of ChemR23, a chemokine receptor that is expressed on immature DCs, and high expression of CCL19, towards which mature DCs migrate, reflect the activation status of pDCs in patients with SLE [30,31,32]. Compared with pDCs from healthy subjects, circulating pDCs from SLE patients stimulated T cells more vigorously; however, they failed to induce Treg cells in the presence of apoptotic cells derived from polymorphonuclear cells [33]. Early depletion of pDCs in lupus-prone BXSB.DTR mice reduced lupus symptoms and signs including organomegaly, reactivity of T and B cells, production of autoantibodies, and severity of kidney pathology, supporting a pivotal role for pDCs in SLE [34].

In SLE, the disruption of innate tolerance to self-antigens is considered to be caused by the formation of ICs. Circulating ICs, containing self-nucleic acids and autoantibodies, trigger the activation of pDCs in SLE [35]. Self-DNA or RNA- containing ICs bind to the low-affinity Fc receptor for IgG (FcγRIIA, also known as CD32) and are then taken up into endosomes as a result of the engagement of TLR9 or TLR7 expressed in pDCs respectively [36]. Lupus-prone mice deficient in TLR7 exhibited an amelioration of the disease including a decrease in serum autoantibody production (e.g., anti-Sm/RNP autoantibody), negative regulation of lymphocyte activation, and reduced severity of lupus nephritis [37]. Overexpression of *Tlr7* gene induced SLE-like phenomena in mice, suggesting that self-RNA-triggered TLR7 signaling contributes to the development of SLE [38]. On the other hand, TLR9 deficient lupus-prone mice exhibited an exacerbation of the disease with increased serum levels of IgG and IFNα [37]. The discordance in the contribution to the development of SLE between TLR7 and TLR9 remains poorly understood. In pDCs, TLR7/9 signaling is propagated via a MyD88-dependent pathway. MyD88 engagement ultimately leads to the expression of Type I/III IFN, proinflammatory cytokines such as IL-6 and TNF-α, and costimulatory molecules including CD40, CD80, and CD86 through the IRF5/7, NFκB, and MAPK pathway [39]. In particular, IRF7 is regarded as a master regulator of Type I IFN production [40]. The involvement of neutrophil has also been emphasized in TLR-7/9-mediated pDC activation in SLE. Compared with the neutrophils of healthy controls, those of SLE patients have a tendency to undergo more neutrophil death by neutrophil extracellular trap (NET) formation referred to as NETosis [41]. Increased NETosis in SLE is caused by ICs or IFNα, and it also results in an increased production of IFNα via pDC activation [41]. NETs extruded by neutrophils contain DNA as well as large amounts of LL37 and high-mobility group box 1 protein (HMGB1) [41,42]. Self-DNA uptaked by LL37, which is an antimicrobial peptide that is abundant in NETs released from neutrophils, can induce pDC activation via TLR9 [41,43]. HMGB1 released by damaged cells binds to nuclear DNA and enhances pDC activation through a TLR9-MyD88- dependent pathway [42].

### 3.2. Role of Type I IFN in SLE

The increased production of type I IFN by pDCs is involved in the pathogenesis of SLE [44]. IFNα is the most important type I IFN in SLE [45]. IFNα therapies sometime induce lupus-like phenomena in patients with malignancy or chronic hepatitis C [46,47]. The level of serum IFNα has been reported to have a positive correlation with lupus disease activity, in some patients [48]. Patients with SLE exhibit an IFN signature, a characteristic expression pattern of type I IFN-inducible genes, in peripheral leukocytes, that is associated with disease activity and severity [44,49,50]. T and B cell lymphopenia frequently found in SLE is sometimes explained by high level of IFNα [51]. Type I IFNs have also been reported to be associated with severe features of SLE such as positivity for anti-dsDNA and presence of nephritis by lowering serotonin synthesis [52].

**Figure 1:**
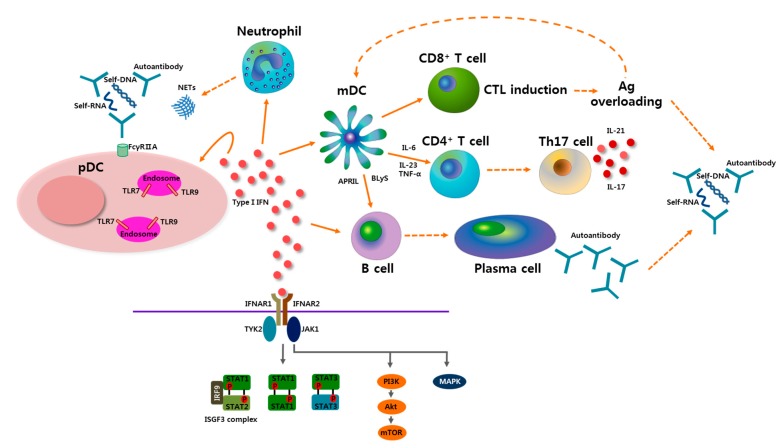
Potential role of plasmacytoid dendritic cells (pDCs) and type I interferon (IFN) in the pathogenesis of systemic lupus erythematosus (SLE). Self-nucleic acids-containing immune complexes activate pDCs by transferring to endosomes after engagement of TLR7 or TLR9. Neutrophil extracellular traps (NETs), which are shed from neutrophils, induce activation of pDCs. Activated pDCs produce profuse type I IFN, which plays a central role in the pathogenesis of SLE. Type I IFN has an effect on many types of immunologic cells, resulting in diverse outcomes. Type I IFN lowers the activation threshold of T cells and B cells by aiding myeloid DCs (mDCs) to produce various stimulators including B lymphocyte stimulator (BLyS), a proliferation-inducing ligand (APRIL), interleukin-6 (IL-6), IL-23, and tumor necrosis factor-α (TNF-α). Th17 cell differentiation, which is supported by type I IFN, has an important role in SLE pathogenesis. IL-17 released by Th17 cells is involved in B cell hyperresponsiveness, autoantibody production, and target organ damage. IL-21, Th17-related cytokine, is also involved in B cell reactivity and follicular helper T cell development. Cytotoxic T lymphocyte (CTL) induction by type I IFN leads to overloading of antigen (Ag), which can be presented by mDCs to other immune cells, and also be a cause of immune complex formation as well as autoantibody production in SLE. After type I IFN binds to type I IFNα receptor (IFNAR), which is a heterodimeric receptor composed of IFNAR1 and IFNAR2, multiple downstream signaling pathways can be involved. Upon activating tyrosine kinase 2 (TYK2) and Janus activated kinase 1(JAK1), various signal transducer and activator of transcription (STAT) proteins are phosphorylated and form heterodimers or homodimers. A canonical IFN-stimulated gene factor 3 (ISGF3) signaling complex, which is a complex of STAT1-STAT2-IFN-regulator factor 9 (IRF9), leads to the induction of IFN-stimulated genes. Other STAT dimers also activate downstream molecules, and they are partially involved in inflammatory responses. Type I IFN signals can also propagate through the mitogen-activated protein kinase (MAPK) and phosphoinositide 3-kinase (PI3K) signaling pathways.

Several mechanisms have been suggested for the pathogenic role of type I IFNs in the pathogenesis of SLE as shown in Figure 1. ICs containing self-nucleic acids, one of the hallmarks of SLE, activate DCs, thus producing abundant type I IFNs. Type I IFNs produced by pDCs stimulate DCs to initiate and maintain their maturation, which is essential for aberrant immune reactions in SLE [53]. Type I IFNs contribute to the generation of both CD4+ and CD8+ T cell responses via the activation of DCs [54,55]. Serum derived from the patients with SLE has increased T cell stimulatory function in mixed lymphocyte reaction study and the stimulatory function was dependent on serum level of IFNα, suggesting the ability of IFNα for stimulating autoreactive T cells [53]. Type I IFNs also promote the cytolytic activities of NK cells and cytotoxic T cells, which could increase tissue damage and antigen overloading, one of major contributors in the development of SLE [56,57]. Type I IFNs increase the production of B lymphocyte stimulator (BLyS) and a proliferation-inducing ligand (APRIL), which are involved in the survival of autoreactive B cells, by mDCs, and this contributes to B cell differentiation and Ig class switching, which are important for generating pathogenic autoantibodies in SLE [7,58,59]. Type I IFNs are also involved in the pathogenesis of SLE by supporting Th17 responses [60]. Type I IFNs promote Th17 responses by inducing the production of IL-6 and IL-23 by DCs [59]. IL-17, produced as a result of Th17 responses, alone or in conjunction with BLyS, can induce B cell hyperreactivity and differentiation into antibody-producing cells [61]. IL-17 can also cause tissue inflammation and organ damage by recruiting neutrophils, lymphocytes and macrophages [62]. In addition, Th17-associated cytokine, IL-21, contributes to breach of immune tolerance in SLE by promoting B cell responses and also by playing a role in the development of follicular helper T cell [63,64].

The analysis of the tissue from SLE patients provides direct evidence of a pathogenic role for type I IFNs in SLE. Synovial tissue from patients with SLE showed characteristic molecular signature, featured by the upregulation of IFN-inducible genes [65]. The IFN signature has also been observed in glomerular tissue from patients with SLE [66]. In a murine model of lupus, IFNα accelerated murine SLE and promoted the development of nephritis [67]. Type I IFNs caused glomerulosclerosis by inducing podocyte death while suppressing renal progenitor differentiation into mature podocytes [68]. Additional data about the effect on cardiovascular system support a pathogenic role for type I IFNs in SLE. Type I IFNs skewed the balance between vascular endothelial cell damage and repair, which led to the acceleration of atherosclerosis in both murine and human SLE [69,70]. A contribution of IFNα to the central nervous system manifestations of SLE is supported by the finding that cerebrospinal fluid from patients with neuropsychiatric lupus had a potency to induce large amounts of IFNα [71].

## 4. pDCs-Type I IFN Axis as a Therapeutic Target in SLE

Nucleic acid-sensing TLRs in pDCs and their product, type I IFN, have become major targets in the development of new therapeutic agents for SLE. Sifalimumab (MEDI-545), a fully humanized IgG1 monoclonal antibody against IFNα, exhibited good tolerability and inhibited the type I IFN signature in some patients with SLE [72,73]. Another humanized IgG1 monoclonal antibody against IFNα, rontalizumab (RG7415), demonstrated an acceptable safety profile, although further investigation is required to determine the clinical efficacy in patients with SLE [74]. Type I IFN priming in SLE was attempted using an IFNα kinoid (IFN-K), which is a synthetic compound composed of IFNα and a carrier protein, keyhole limpet hemocyanin [75]. Active immunization with IFN-K resulted in the production of anti-IFNα antibodies and improved disease activity markers in patients with SLE [75]. A few attempts at modulating TLR signaling in SLE suggest both promising safety and efficacy profiles [76].

## 5. Conclusions

Activation of the pDCs-type I IFN axis plays a central role in the pathogenesis of SLE, as evidenced by multiple experimental and observational studies. Although pDCs-type I IFN axis-based therapies for treating SLE are an opening stage, a better understanding of this axis in SLE would potentially enhance the development of new therapeutic agents for the management of SLE.
